# Determinants of first-time utilization of long-term care services in the Netherlands: an observational record linkage study

**DOI:** 10.1186/s12913-017-2570-z

**Published:** 2017-09-05

**Authors:** Laurentius C.J. Slobbe, Albert Wong, Robert A. Verheij, Hans A.M. van Oers, Johan J. Polder

**Affiliations:** 1 National Institute for Public Health and the Environment (RIVM), RIVM, PO Box 1, 3720 BA Bilthoven, The Netherlands; 2 Tilburg University, Tranzo, Tilburg School of Social and Behavioral Sciences, PO Box 90153, 5000 LE Tilburg, The Netherlands; 3 Netherlands Institute for Health Services Research (NIVEL), NIVEL, PO box 1568, 3500 BN Utrecht, The Netherlands

**Keywords:** Long-term care utilization, Chronic disease, Administrative data, Modelling

## Abstract

**Background:**

Since in an ageing society more long-term care (LTC) facilities are needed, it is important to understand the main determinants of first-time utilization of (LTC) services.

**Methods:**

The Andersen service model, which distinguishes predisposing, enabling and need factors, was used to develop a model for first-time utilization of LTC services among the general population of the Netherlands. We used data on 214,821 persons registered in a database of general practitioners (NIVEL Primary Care Database). For each person the medical history was known, as well as characteristics such as ethnicity, income, home-ownership, and marital status. Utilization data from the national register on long-term care was linked at a personal level. Generalized Linear Models were used to determine the relative importance of factors of incident LTC-service utilization.

**Results:**

Top 5 determinants of LTC are need, measured as the presence of chronic diseases, age, household size, household income and homeownership. When controlling for all other determinants, the presence of an additional chronic disease increases the probability of utilizing any LTC service by 45% among the 20+ population (OR = 1.45, 95% CI: 1.41–1.49), and 31% among the 65+ population (OR = 1.31, 95% CI: 1.27–1.36). With respect to the 20+ population, living in social rent (OR = 2.45, 95% CI = 2.25–2.67, ref. = home-owner) had a large impact on utilizing any LTC service. In a lesser degree this was the case for living alone (OR = 1.63, 95% CI = 1.52–1.75, ref. = not living alone). A higher household income was linked with a lower utilization of any LTC service.

**Conclusions:**

All three factors of the Anderson model, predisposing, enabling, and need determinants influence the likelihood of future LTC service utilization. This implies that none of these factors can be left out of the analysis of what determines this use. New in our analysis is the focus on incident utilization. This provides a better estimate of the effects of predictors than a prevalence based analysis, as there is less confounding by changes in determinants occurring after LTC initiation. Especially the need of care is a strong factor. A policy implication of this relative importance of health status is therefore that LTC reforms should take health aspects into account.

**Electronic supplementary material:**

The online version of this article (10.1186/s12913-017-2570-z) contains supplementary material, which is available to authorized users.

## Background

The cost of long-term care (LTC), defined as care for people needing daily living support over a prolonged period of time [1], is on the rise in many countries. In 2013, an average 1.1% of Gross Domestic Product (GDP) was spent on health related LTC in OECD- countries, with a large variation [2]. The reported utilization of these services ranges from <0.1% of GDP in the Slovak republic, to 2.9% of GDP in the Netherlands. Insight into what determines LTC utilization may help in understanding what drives the rise in LTC use.

According to Andersen’s health care utilization model [3], determinants of LTC can be classified into three groups: predisposing, enabling and need determinants. The most important predisposing variables are age, time to death, and in some countries race or ethnicity [4–6]. Enabling determinants include available support within the context of family or community. For example, living alone or having a small social network is associated with a higher demand for LTC services [7]. Also income, wealth, homeownership and socio-economic status belong to this category, but these factors influence LTC use in a more complex manner depending on the way in which countries have organised the provision of long-term care [8]. Finally, there are need determinants, which refer to the more or less ‘objective’ need for LTC based on someone’s physical or mental condition.

Distinguishing these three categories of determinants raises the question about their relative importance, and the manner in which they are related. On the one hand, given the fact that a limitation in daily functioning is often a requirement for gaining access to LTC services, it is to be expected that limitations in Activities of Daily Living (ADL), which in many studies figure as some form of health status or disability measure (i.e. a need determinant), are a good predictor of LTC utilization [9].

On the other hand, even though health status plays an obvious role in admittance to LTC, the effect of health on actual utilization might be swamped by that of poverty or lack of social support. Alternatively, the influence of health might already be captured by the predisposing variable age. The study we present here was undertaken to evaluate the relative contributions of these categories of determinants and to unravel their interrelatedness. However, the underlying data sets available for analysis need to be rich enough in order that quantitative assessment of the most important determinants within each category can be done. In this respect, the healthcare system of the Netherlands offers a potentially fertile field for addressing the task we set ourselves, as it includes, amongst others, a nation-wide system of institutionalized LTC with a comprehensive system of administration. Moreover, these data are linkable at an individual level to medical records and to various socio-economic parameters.

Making use of the availability of such comprehensive data, we further explored a novel approach to avoid what we perceive as a short-coming in many other studies.

Most studies so far focused on determinants of current use of LTC. This has the disadvantage that key determinants such as income, wealth and health status are likely to have changed since the start of LTC-use. For example, a heart condition in a current LTC-user might well have developed since entry into LTC-use, where LTC-use itself may have adversely affected wealth or living conditions.

The aim of this study is to develop a prediction model for long-term care using determinants collected from administrative datasources. In order to avoid the confounding that results from using current use of LTC we have explored a novel approach in which we looked at first-time use of LTC-services. The hypothesis is that focusing on first time users of LTC allows for identifying determinants of LTC care that are more directly and ‘causally’ associated with LTC use. As a secondary aim we investigated if it was possible to replace the most commonly encountered need determinant in literature (ADL) with another, more easily calculated from administrative sources.

## Methods

### Study population and data

The core of the dataset we used consisted of a large primary care database with information on chronic diseases as registered by general practitioners (GP), maintained by the Netherlands Institute for Health Services Research (NIVEL-PCD). More than 500 general practices participate in NIVEL-PCD, 10% of all Dutch general practices. Since all Dutch citizens are registered at a GP, our sample is largely representative for the Dutch population. We linked these data at a personal level with the national LTC-register, with data on LTC-use for the entire 20+ population, and with data on predisposing and enabling factors from several data sources available at Statistics Netherlands (CBS). Thus an artificial cohort was constructed (Table 1). Due to gaps in the LTC register before 2008 we could only use data from 2008 to the latest available year (2011). The remaining dataset consisted of 214,821 persons of age 20+ in 2008–2011. As an uninterrupted stretch of three years of contact data is deemed necessary to be able to establish the presence or absence of chronic disease, we analysed incident LTC-utilization in 2011 (see below) according to the determinants in 2008–2010. Health status (NIVEL-PCD as data source) was measured by the number of chronic diseases present at December 31st 2010. Whereas chronic diseases were defined using a list provided by O’Halloran [10]. This list had been successfully applied earlier to NIVEL-PCD-data [11]. See Additional file 1 for the full list. A patient was counted as prevalent for a chronic disease if at least one contact for this disease was registered during 2008–2010. The count of the number of diseases per patient varied between zero and nine.

**Table 1:** Selection of the study population

Selection made	Explanation	Number of subjects after selection
1: all citizens registered for at least one year in GP-database between 1-1-2006 and 1–1-2011	all potentially eligible subjects in artificial cohort.	701,790
2: all patients registered for a continuous period of at least three years in GP-database	three year continuous registration necessary for establishing disease prevalence.	441,809
3: all patients alive at 1–1-2012	necessary for full follow-up in LTC register for at least one year after last recorded contact date in GP-database	427,115
4: all patients with complete medical records for 2008–2010	restriction of the analysis to GP-data 2008–2010 was necessary because LTC-utilization database was incomplete for 2007	276,721
5: removal of persons with missing age	three cases were removed because a subselection on age had to be made	276,718
6: restriction to persons 20+ of age at 1–1-2011	as LTC register covered only adult population, GP-dataset was restricted likewise.	214,821

Age, gender, ethnicity and household type were assessed on December 31st 2010, and home-ownership, household income and assets were determined for the year 2010 (Statistics Netherlands administrative databases as datasource). For ethnicity a classification adapted to the Dutch population was used. The main distinction is made on migration history. We have lumped different countries of origin with the same migration history together in one group, as is common in studies of the Dutch population. After the native Dutch the largest groups are formed by labour migrants from islamic countries (Morocco or Turkey) and by migrants from (former) Dutch Caribbean possessions (Surinam or Dutch Antilles). If a subject has at least one grandparent with a migrant background, he or she is included in this group.

Long-term care utilization was defined as the use (yes/no) of formal LTC services provisioned by Dutch healthcare organizations (National LTC-register as data source). An extensive overview of the Dutch system for LTC provision as of 2010 can be found in Mot [12]. A total of six different (combinations of) LTC services were distinguished. Table 2 provides a more detailed description, and links the Dutch LTC services to the health functions as used in the System of Health Accounts (SHA) of the OECD [13]. We counted utilization as incident if someone utilized LTC in 2011, but did not utilize any LTC service in the years 2008–2010. Patients with any previous utilization of LTC services in 2008–2010 were removed from the dataset, resulting in a study population of 197,100 cases.

**Table 2:** Types of LTC- services used in the analysis, based on the OECD System of Health Accounts [13]

Type of long-term care service	Included in SHA 2011 function	Description
LTC residential	HC.3.1, HC.3.2	Care provided in a residential long-term care facility requiring medical supervision, mostly with overnight stay (HC.3.1). Day-care (HC.3.2) is also included.
LTC home: nursing	HC3.4	Health services provided to persons within their own home. It can involve specialised health care and requires the assistance of medical professionals.
LTC home: personal	HC3.4	Services provided in kind and at home to assist with activities of daily living related to personal hygiene like bathing or dressing.
LTC home: domestic	HCR.1.1	Social care services provided in kind and at home to assist with instrumental activities of daily living, such as domestic cleaning.
LTC home: any type	HC.3.4, HCR.1.1	Home care of any type: nursing, personal or domestic care.
LTC home or residential	HC.3.1, HC.3.2, HC.3.4, HCR.1.1	Any of the LTC-types described above.

#### Activities of daily living (ADL) limitations versus number of chronic diseases

In many studies limitations in ADL were used as measure of health status, instead of the number of chronic diseases we used in our study [4, 9]. In order to analyse the difference we retrieved for a subset of 2814 subjects data on ADL disability, measured on a four-point scale (1–4), ranging from no limitation to severe limitation (Statistics Netherlands Household Survey as data source). This sample, however, was too small to be used as a need determinant in the main analysis.

### Statistical analysis

Logistic regression was used to model the relation between the incident LTC service utilization in 2011 and the available predictors in 2008–2010. Several alternative operationalizations were available for some predictors. For instance, ‘household income’ could be operationalized as gross income, income after taxes or income standardized for household. In order to make an optimal choice between these alternatives, they were first tested in single-predictor models. The best-performing predictor or set of predictors were then chosen, as evaluated on the basis of the Akaike information criterion (AIC, see Additional file 2 for values), which penalizes models that are overspecified.

The final model was constructed by combining the best-performing predictors with a multivariate model. Table 3 lists a description of outcome variables and predictors used in the models.

**Table 3:** Variables used in the LTC service-utilization models

Variable	Type	Description
LTC Outcome		
*LTC home or residential*	binomial	1 = Long-term care utilization of any type in 2011, 0 = no utilization of any type
*LTC residential*	binomial	idem, for residential care only
*LTC home: any type*	binomial	idem, home care of any type
*LTC home: domestic*	binomial	idem, home care: house cleaning
*LTC home: nursing*	binomial	idem, home care: nursing care
*LTC-home: personal*	binomial	idem, home care: personal care
Predisposing determinants		
*Gender*	categorical	Male = 0 (reference), Female = 1
*Centered age*	numerical	Based on Age at December 31st, 2010. Computed as: (Age – average (Age))/10.
*Centered age .^2*	numerical	Computed as square of centered age
*Origin*	categorical	Country of origin. If subject or at least one parent is foreign-born, subject is classified using foreign country. Otherwise classified as native Dutch. 0 = native Dutch (reference), 1 = Morocco or Turkey, 2 = Surinam or Dutch Antilles, 3 = other western, 4 = other non-western
Enabling determinants		
*Gross household income*	numerical	Gross household income in 2010 divided by 10,000 euro
*Single person household*	categorical	1 = living alone December 31st 2010, 0 = otherwise (reference)
*Housing situation*	categorical	Housing situation at December 31st 2010: 0 = Homeowner (reference), 1 = Social rent, 2 = Private rent.
Need determinants		
*Nr. of chronic diseases*	numerical (integer)	Number of prevalent chronic diseases: diseases with at least one GP contact in 2008–2010
*ADL classification*	categorical	Classification activities of daily living, as used in Dutch housing survey: 0 = no limitation (reference), 1 = light limitation, 2 = some limitation, 3 = severe limitation

The regression model can be specified as follows:

$$ \mathrm{logit}\left({p}_i\right)=\ln \left({p}_i/\left(1-{p}_i\right)\right)={\upbeta}_0+{\upbeta}_1{\mathrm{x}}_{1,\mathrm{i}}+\dots {\upbeta}_{\mathrm{m}}{\mathrm{x}}_{\mathrm{m},\mathrm{i}} $$

In which *p*_*i*_ represents our LTC-service outcome variables, x_1,i_ + … x_m_ the predictors and β_0_ … β_m_ the model coefficients.

This model was applied successively to all six LTC-service outcome variables as the dependent variable. Although most users of LTC are 65+ of age, first-time use often starts earlier. We decided to run the model separately for 20+ and 65+ populations, so we could evaluate if this would result in markedly different models. The performance of the models was estimated using *k*-fold cross-validation, with k = 10 [14]. All subjects in the artificial cohort were randomly assigned to one of the 10 folds, and for each fold a seperate prediction was made based on the data of the other nine folds. From these predictions average specificity, sensitivity, accuracy and negative predictive value were estimateded.

#### ADL limitations versus number of chronic diseases

As mentioned above, our study used a need determinant – the number of chronic diseases – that is less common used than the ADL disability score. We estimated what difference it would have made if we had used ADL limitations rather than number of chronic diseases, as follows. Three additional models were defined for the small subset of data for which we had both an ADL disability score and information on the number of diseases present (see above). One model included both the ADL disability score and the number of chronic diseases present as need determinants, while the other two models included only one of these. All predisposing and enabling determinants utilized in analysis of the full set were included in these models.

## Results

Table 4 provides an overview of the data used, the variables analysed, and the distribution of the studied population over these variables.

**Table 4:** Study population

	Proportion, average or count	Proportion (%) of first-time users of LTC services in 2011
All subjects (N)	214,821	2.2
Predisposing determinants		
Gender (%) (*N* = 214,818)		
*Male*	47.1	2.0
*Female*	52.9	2.4
Age (average)	50.8	
Age group (%) (*N* = 214,818)		
*20–44*	38.0	1.1
*45–64*	39.6	1.3
*65–84*	19.9	6.0
*85+*	2.5	23.4
Country of origin (%) (*N* = 214,818)		
*Autochthonous (Native Dutch)*	82.9	2.3
*Morocco + Turkey*	3.1	1.7
*Surinam + Dutch Antilles*	2.5	2.1
*Other non-western*	2.8	1.4
*Other western*	8.6	2.3
Need determinants		
Nr. of chronic diseases with at least one GP contact in 2008–2010		
*0*	64.6	1.2
*1*	23.5	3.3
*2*	8.0	6.1
*3*	2.7	9.2
*4*	0.88	13.5
*> =5*	0.38	16.8
ADL-score (for subset of 2814 subjects) (%)		
*1 = no limitation*	85.6	3.4
*2 = light limitation*	3.7	17.1
*3 = some limitation*	8.7	32.0
*4 = severe limitation*	2.0	56.4
Enabling determinants		
Household type (%) (*N* = 214,393)		
*Single person*	18.4	5.3
*Otherwise*	81.6	1.6
Gross household income (*N* = 213,665)		
*Before taxes (€ × 1000, average)*	63.8	
Home ownership (%) (*N* = 213,665)		
*Home owner*	67.7	1.3
*Rent with social security assistance*	10.3	6.4
*Other rent*	21.9	3.2

Legend: First column describes the variable, second column gives the value. The third column lists the proportion of first-time users of LTC in 2011. This was calculated as the proportion of subjects who utilized any form of LTC in 2011, but did not utilize any type of LTC in 2008–2010. Prevalent cases (with any prior use of LTC in 2008–2010) were excluded from both numerator and denominator

Results from the statistical analysis are shown in three tables. Table 5 shows results from the logistic regression for the 20+ population, Table 6 for the 65+ population, and Table 7 for the three models used in verifying the effect of substituting the more common ADL disability score for our need determinant. Outcomes for the different determinants are presented as odds-ratios (OR) with 95% confidence intervals. All OR given are adjusted for the influence of the other determinants. In Additional file 3 full results for all models can be found. In Additional file 4, results of the cross-validation exercise are listed.

**Table 5:** Model outcomes for 20+ population for all LTC services

Ages: 20+ (*N* = 197,100)	LTC-home or residential	LTC- residential	LTC-home: any type	LTC-home: domestic	LTC-home: nursing	LTC-home: personal
Model (adjusted OR except for constant)						
Constant	−4.595	−6.903	−4.649	−6.551	−6.872	−6.784
*Predisposing determinants*						
Female (ref = male)	1.11 (1.04–1.18)	0.88 (0.75–1.03)	1.13 (1.05–1.20)	2.10 (1.84–2.39)	1.00 (0.88–1.14)	1.12 (1.00–1.25)
Centered age	1.04 (1.01–1.07)	1.09 (0.99–1.19)	1.04 (1.01–1.08)	1.54 (1.41–1.67)	1.88 (1.66–2.12)	1.87 (1.67–2.09)
Square of centerd age	1.12 (1.11–1.13)	1.18 (1.16–1.21)	1.11 (1.10–1.12)	1.05 (1.02–1.07)	1.03 (1.00–1.06)	1.08 (1.06–1.10)
Origin: Morocco + Turkey (ref = autochtonous)	0.79 (0.65–0.96)	0.50 (0.22–1.12)	0.81 (0.66–1.00)	0.92 (0.61–1.40)	1.07 (0.62–1.85)	0.80 (0.46–1.41)
Origin: Surinam + Dutch Antilles	0.89 (0.73–1.09)	1.14 (0.66–1.96)	0.88 (0.72–1.08)	0.68 (0.44–1.05)	0.72 (0.40–1.32)	0.59 (0.34–1.03)
Origin: other western	0.88 (0.78–0.98)	0.72 (0.53–0.98)	0.90 (0.81–1.01)	0.81 (0.66–0.99)	0.96 (0.77–1.21)	0.82 (0.67–1.00)
Origin: other non-western	0.66 (0.53–0.82)	0.54 (0.24–1.23)	0.69 (0.55–0.86)	0.73 (0.46–1.14)	0.87 (0.47–1.59)	0.60 (0.32–1.13)
*Enabling determinants*						
Single person houshold = yes (ref = no)	1.63 (1.52–1.75)	1.46 (1.23–1.74)	1.66 (1.54–1.79)	1.89 (1.67–2.15)	1.20 (1.02–1.41)	1.50 (1.33–1.70)
Gross household income	0.91 (0.90–0.92)	0.92 (0.89–0.94)	0.91 (0.90–0.92)	0.87 (0.85–0.89)	0.98 (0.96–1.00)	0.94 (0.92–0.96)
House: Social rent (ref = home-owner)	2.45 (2.25–2.67)	1.61 (1.28–2.01)	2.49 (2.28–2.72)	2.56 (2.21–2.97)	1.21 (1.00–1.47)	1.26 (1.08–1.47)
House: Private rent	1.51 (1.40–1.63)	1.73 (1.44–2.08)	1.47 (1.35–1.59)	1.45 (1.25–1.68)	1.14 (0.98–1.34)	1.10 (0.97–1.26)
*Need determinants*						
Nr of chronic diseases	1.45 (1.41–1.49)	1.30 (1.21–1.38)	1.45 (1.41–1.50)	1.36 (1.29–1.42)	1.43 (1.36–1.51)	1.39 (1.33–1.45)

Legend: Odds values (with 95% confidence interval) are presented except for the constant. For numerical values, odds denote the effect of the increase of one unit; for categorical variables they denote the effect of a change from the reference category to the designated category

**Table 6:** Model outcomes for 65+ population for all LTC services

Ages: 65 + (*N* = 35,615)	LTC-home or residential	LTC- residential	LTC-home: any type	LTC-home: domestic	LTC-home: nursing	LTC-home: personal
Model (adjusted OR except for constant)						
Constant	−7.025	−9.871	−7.253	−10.176	−6.630	−7.946
*Predisposing determinants*						
Female (ref = male)	1.23 (1.12–1.35)	0.99 (0.82–1.20)	1.24 (1.12–1.36)	2.04 (1.74–2.39)	0.98 (0.83–1.16)	1.09 (0.96–1.24)
Centered age	4.07 (2.21–7.50)	6.51 (1.90–22.28)	4.55 (2.41–8.59)	15.07 (5.44–41.76)	2.01 (0.70–5.81)	3.90 (1.70–8.92)
Square of centerd age	0.95 (0.87–1.03)	0.91 (0.77–1.08)	0.93 (0.85–1.01)	0.76 (0.66–0.88)	1.01 (0.87–1.17)	0.97 (0.87–1.09)
Origin: Morocco + Turkey (ref = autochtonous)	0.72 (0.39–1.30)	0.00 (0,∞)	0.79 (0.43–1.42)	0.75 (0.30–1.84)	0.65 (0.20–2.04)	0.29 (0.07–1.18)
Origin: Surinam + Dutch Antilles	0.67 (0.41–1.08)	1.19 (0.52–2.72)	0.53 (0.30–0.91)	0.39 (0.16–0.95)	0.72 (0.30–1.77)	0.57 (0.27–1.23)
Origin: other western	0.94 (0.81–1.09)	0.79 (0.56–1.11)	0.98 (0.84–1.14)	0.73 (0.56–0.94)	0.86 (0.65–1.14)	0.84 (0.67–1.05)
Origin: other non-western	0.57 (0.30–1.09)	0.69 (0.17–2.82)	0.63 (0.33–1.19)	0.50 (0.18–1.37)	0.95 (0.35–2.58)	0.42 (0.13–1.34)
*Enabling determinants*						
Single person houshold = yes (ref = no)	1.39 (1.26–1.54)	1.48 (1.20–1.81)	1.41 (1.27–1.56)	1.66 (1.43–1.94)	1.11 (0.92–1.34)	1.44 (1.25–1.65)
Gross household income	0.94 (0.92–0.96)	0.97 (0.93–1.02)	0.94 (0.92–0.96)	0.88 (0.85–0.92)	0.96 (0.93–1.00)	0.96 (0.93–0.99)
House: Social rent (ref = home-owner)	1.46 (1.30–1.65)	1.22 (0.94–1.59)	1.48 (1.31–1.67)	2.06 (1.72–2.46)	1.12 (0.90–1.40)	1.15 (0.97–1.36)
House: Private rent	1.22 (1.11–1.36)	1.59 (1.30–1.96)	1.16 (1.04–1.29)	1.36 (1.14–1.61)	1.04 (0.87–1.26)	1.02 (0.88–1.17)
*Need determinants*						
Nr of chronic diseases	1.31 (1.27–1.36)	1.24 (1.15–1.33)	1.31 (1.27–1.36)	1.22 (1.16–1.30)	1.35 (1.27–1.43)	1.31 (1.25–1.38)

Legend: Odds values (with 95% confidence interval) are presented except for the constant. For numerical values, odds denote the effect of the increase of one unit; for categorical variables they denote the effect of a change from the reference category to the designated category

**Table 7:** Model outcomes for the 20+ population with established ADL for all LTC services

Ages 20+ with known ADL-score *N* = 2814			
Outcome: LTC-home or residential			
	model-1	model-2	model-3
Model (adjusted OR except for constant)			
Constant	−4.422	−4.406	−4.186
*Predisposing determinants*			
Female (ref = male)	0.82 (0.47–1.46)	0.74 (0.41–1.33)	0.73 (0.41–1.29)
Centered age	1.11 (0.84–1.47)	1.08 (0.81–1.44)	1.16 (0.88–1.53)
Square of centerd age	1.08 (1.00–1.17)	1.08 (1.00–1.17)	1.08 (0.99–1.17)
Origin: Morocco + Turkey (ref = autochtonous)	0.45 (0.06–3.49)	0.35 (0.04–2.84)	0.35 (0.04–2.75)
Origin: Surinam + Dutch Antilles	0.49 (0.06–3.82)	0.45 (0.06–3.49)	0.42 (0.05–3.22)
Origin: other western	0.39 (0.12–1.30)	0.37 (0.11–1.25)	0.40 (0.12–1.32)
Origin: other non-western	1.38 (0.30–6.32)	1.17 (0.25–5.49)	1.16 (0.25–5.41)
*Enabling determinants*			
Single person houshold = yes (ref = no)	1.89 (0.99–3.60)	1.86 (0.98–3.54)	1.74 (0.92–3.28)
Gross household income	0.86 (0.73–1.01)	0.86 (0.74–1.02)	0.86 (0.73–1.01)
House: Social rent (ref = home-owner)	2.69 (1.23–5.91)	2.69 (1.23–5.92)	2.92 (1.33–6.39)
House: Private rent	1.37 (0.65–2.86)	1.34 (0.64–2.81)	1.38 (0.67–2.88)
*Need determinants*			
Number of chronic diseases	1.46 (1.13–1.88)	1.41 (1.09–1.82)	-
ADL-score	-	1.37 (0.99–1.91)	1.47 (1.06–2.03)

Legend: All models contain the same determinants, with the exception of the need determinants. Model-1 contains the number of chronic diseases as need determinant, Model-3 contains the ADL-score. Model-2 contains both available need determinants. Odds values (with 95% confidence interval) are presented except for the constant. For numerical values, odds denote the effect of the increase of one unit; for categorical variables they denote the effect of a change from the reference category to the designated category

### Predisposing determinants

As expected, age was a significant predictor in almost all models. In the 20+ population models, second-order age effects (age*age) are also significant; in most 65+ population models only first-order effects of age are significant. Only for LTC home-care nursing services age is not a significant predictor in the 65+ population. Females had a much higher probability of first-time use of any LTC-service than males, especially LTC home-care services. The odds of incident utilization of domestic LTC- services is twice as large for women (for 20+: OR = 2.10, 95% CI: 1.84–2.39, for 65+: OR = 2.04, 95% CI: 1.74–2.39).

Among the 20+ population being of non-Dutch origin was associated with a decreased likelihood of first-time utilization of LTC services. LTC-home or residential service was lowest for citizens born in Morocco or Turkey (OR = 0.79, 95% CI: 0.65–0.96, with the autochthonous population as reference). Among the elderly (65+) country of origin was not significant due to low numbers of people in these groups.

### Enabling determinants

Living alone increased the proportion of first-time users of any LTC-home or residential service compared to living in a multi-person household, in both the 20+ (OR = 1.63, 95% CI: 1.52–1.75) and 65+ population (OR = 1.39, 95% CI: 1.26–1.54). The odds were higher for the use of domestic home-care services in both the 20+ (OR = 1.89, 95% CI: 1.67–2.15) and 65+ populations (OR = 1.66, 95% CI: 1.43–1.94).

Wealth was represented in our models by income and asset measures. A higher gross household income was associated with a decrease in first-time utilization of LTC services. For the first-time utilization of any type of LTC-service, an increase of 10,000 euro is associated with an OR = 0.91 (95% CI: 0.90–0.92) for the 20+ population and an OR = 0.94 (95% CI: 0.92–0.96) for the 65+ population. For LTC domestic home-care services the income effect is somewhat stronger for both the 20+ (OR = 0.87, 95% CI: 0.85–0.89) and 65+ population (OR = 0.88, 95% CI: 0.85–0.92). The best performing asset measure proved to be a classification of home-ownership into three groups: ‘homeowners’ (reference category), ‘social rent’ and ‘private rent’. Subjects classified under ‘social rent’ pay lower rents, because they have a low income compared to the market rates and get a tax break to remedy this. Subjects classified under ‘private rent’ pay rents at market prices.

Both types of rent were associated with a higher incident utilization of any type of LTC services. Social renting carries the highest odds (the 20+ population 2.45 (95% CI: 2.25–2.67); in the 65+ population1.46 (95% CI: 1.30–1.65)). The ‘private rent’ group also showed an elevated first-time use of any type of LTC-service compared to homeowners, but less than the social-rent category.

The higher utilization associated with renting a home applies to all types of LTC services, with the exception of personal and nursing care delivered at home, homeownership status not being a significant predictor for this type of care. This in contrast with domestic care services delivered at home, which is strongly associated with social renting (OR = 2.56 (95% CI: 2.21–2.97) for the 20+ population and OR = 2.06 (95% CI: 1.72–2.46) for the 65+ population).

### Need determinants

The number of chronic diseases present was a significant predictor for incident utilization of LTC services in all models. For all types of LTC service combined, the odds ratio (OR) associated with an additional chronic disease was 1.45 (1.41–1.49) for the 20+ population and 1.31 (1.27–1.36) for the 65+ population. In the 20+ population this association seems weakest for residential LTC services, in the 65+ population for both residential LTC services and domestic home-care.

#### ADL versus number of chronic diseases

The ADL disability score and the number of chronic diseases present had similar odds ratios in the separate analysis in which these alternative measures of health status were compared. Due to the small sample we were only able to perform the comparison in the 20+ population with utilization of any type of LTC-home or residential services as the outcome (Table 7). In the model with ADL disability as sole need determinant, a one-point step upwards in the four-point ADL disability scale was associated with OR = 1.47 (95% CI: 1.06–2.03). In the model with the number of chronic diseases present as sole need determinant, an additional chronic disease carried an OR = 1.46 (95% CI: 1.13–1.88). This last value is almost the same as that obtained with the same model on the full set (OR = 1.45, 95% CI: 1.41–1.49, Table 5). This is a strong indication that the sample is representative for the whole set. If we include both measures, only the number of chronic diseases stays significant (OR = 1.41, 95% CI: 1.09–1.82).

### Cross-validation

The full results of the cross-validation for all models are shown in Additional file 4. The sensitivity of the models ranges between 0.59–0.81 and the specificity between 0.62–0.84. All models perform very well in predicting a person not going to use LTC services, with a negative predictive value within the range 0.97–0.99 for all models. However, the precision in predicting who will use LTC services is low, with values of 0.01–0.13. The accuracy of the models was highest in the 20+ dataset, which correctly predict 0.75–0.84 of cases in the cross-validation exercise, but was lower (0.63–0.68) among the 65+ population.

## Discussion

To summarize, all three factors of the Anderson model: predisposing, enabling and need determinants, are of importance when predicting LTC use. An implication for the future study of LTC use is that it is of paramount importance to include data on all these factors in the analysis, like has been done in our study. This does not mean that for each type of LTC service each factor is of equal importance. But just for a single model, LTC home nursing services for the 65+ population, only one factor of the Anderson model is significant, the need determinant ‘number of chronic diseases’. This exception underlines the suprising importance of ‘need’ as a determinant in all models, even for LTC services like domestic cleaning which is often viewed as a social service and not a health service, and therefore not included in many national calculations of the cost of health care [13]. Our study therefore questions the artificial distinction between social services and health services.

In contrast to other studies found in the literature, our study focused on incident LTC use, rather than prevalent LTC use [15, 16]. In this respect, it is important to emphasize that, in contrast to most other studies [17–19], we did not look solely to the older population (65+). In fact, in our study population about a third of all incident cases occurred between the ages of 20–65.

In line with other studies, our study confirms the importance of predictors such as old age and health status, living alone and living in a rented house as contributors to LTC service utilization [4, 8, 20]. Indeed, our analyses further substantiate the protective influence of wealth (home ownership, income) on utilization of LTC services discussed in the literature. We were able to quantify this influence in greater detail than other studies, owing to the large sample size, and the range of determinants for which information was available. In particular, we found that homeownership may even be the most important wealth-related determinant of LTC utilization, as already proposed by Rouwendal and Thomese [8] in a study based on a much smaller dataset. Also the inverse relation between LTC utilization and size of income [21] was confirmed.

### Applicability in LTC projections

An important application of a better understanding of which determinants drive LTC use is in improving predictions of future demand for LTC services. Modelling of future demand for LTC services is often based on demographic projections alone, keeping other factors such as disability prevalences equal [22, 23], or it is based on macro-economic simulation models [1, 22]. These models allow for crude projections of future demand. A more sophisticated model was developed by De Meijer [24], based on the relation between disability level, age and gender. This model has been applied to estimate the effect of ageing on expected LTC costs. The results of our study suggest that including other predictors in such models, such as the number of chronic diseases, homeownership and household size, could substantially improve such projections. For instance, including the number of chronic diseases as a predictor would make it possible to estimate the effects of different epidemiological scenario’s, such as alternative trends in disease prevalence, on future utilization of LTC services.

In this respect, it is of note that the number of prevalent chronic diseases seems to be interchangeable with ADL disability as a predictor of incident utilization of LTC services. Obviously, there is a strong correlation between ADL disability and the presence of chronic diseases, as confirmed by recent studies into the contribution of specific diseases to disability [25–27]. That these alternative indicators of health status have similar predictive power, was clearly shown in the separate analysis we performed on the smaller dataset in comparing them. Our model of LTC utilization will thus have distinct advantages in comparison to the currently available predictive health care utilization models, especially in situations in which no information is available on ADL disability, but there is information on disease prevalence.

To our knowledge, no other model exists for predicting incident utilization of LTC services based on the prevalence of chronic disease. Another possible useful application of this model, besides projecting future demands, is screening a population for the current need of LTC services. It would also be interesting to employ and further develop the model in comparative studies, for instance by applying it to a country with a different long-term care system, relying less on formal services than the Netherlands. Or, alternatively, we might consider the long term effects of current trends in Dutch politics [28], in which access to LTC services is further restricted to people with the most severe disabilities and without assets or social support. It seems likely that this would lead to the need and enabling determinants becoming more strongly associated with LTC use, while the influence of predisposing predictors such as age and gender will decrease.

### Limitations and strengths

For this study we depended on administratively available quantitative data, which comes with some limitations. One of these is that we were not able to capture the utilization of LTC services when initiated by sudden health shocks, e.g. accidents, which do not show up in our selection of chronic conditions.

A further potential limitation concerns the representativeness of our dataset. Our study population consisted of citizens registered at GP practices participating in a network collecting data on primary care. Although large, this is not necessarily a representative sample of the Dutch population. The average age of 20+ study population was almost the same as (50.8) as the average reported by statistics Netherlands for the Dutch 20+ population (49.1) in 2010, but the average gross household income was higher than the Dutch average in 2010 (63,800 euro against 56,100).

Also the proportion of individuals using LTC services was comparable to the Dutch population. Thus 7.85% of our study population used LTC services in 2011, including those with previous utilization, which is close to the 8.46% reported by Statistics Netherlands for the whole Dutch 18+ population in the same year. We conclude that there are no indications of a selection bias that would restrict the generalizability of our findings.

A final and major strength of our study is that determinants and LTC utilization were measured independently of each other. Thus, we can be confident that our need determinant, the prevalence of chronic disease, was estimated in a manner free from an upcoding bias, because this prevalence was calculated from GP data and not known to the administrative bodies that regulate access to the LTC services.

## Conclusions

We demonstrate it is possible to construct a prediction model for the first-time utilization of LTC-services. The models we have built from routinely collected administrative sources might be of use in allocating resources for LTC services, for instance by identifying areas in which future demand in the population will be higher or lower than average. We also concluded that all factors in the Anderson model are relevant. Predisposing, enabling, and need determinants all influence the likelihood of future LTC utilization. This implies that none of these factors can be left out of the analysis of what determines this use. Need, measured as the presence of chronic diseases, age, household size, household income and homeownership are significant predictors. .

Finally, a policy implication of the relative importance of health status in our models is that LTC reforms should also take health aspects into account.

## Additional file

Additional file 1: Selection and definition of chronic diseases used in study. Diseases are defined using the ICPC-1 classification for primary care. (XLSX 12 kb)
Additional file 2: Model selection. Statistical information and description of the model selection process. (XLSX 20 kb)
Additional file 3: Statistical parameters final models. Statistical addendum for models in Tables 5, 6 and 7. Contains coefficients, confidence intervals and *p*-values for all determinants. (XLSX 27 kb)
Additional file 4: Extended versions of Tables 5 and 6, including outcomes for 10-K cross-validation of the models. For each model sensitivity, specificity, threshold, accuracy, precision and negative predictive value are specified. (XLSX 12 kb)
